# Supplementary material for: Convergent mapping of a tremor treatment network
Source: Nat Commun. 2025 May 22;16:4772. doi: 10.1038/s41467-025-60089-6 (PMC12098757; doi:10.1038/s41467-025-60089-6)
Supplement: Supplementary file 1 — Supplementary Information [file 41467_2025_60089_MOESM1_ESM.pdf]

Convergent mapping of a tremor treatment network

(Goede et al., 2025)

Supplementary Information

Table of Contents

Figures

S1 Modeling considerations

S2 Correlation maps for VIM-DBS patients based on follow-up duration

S3 Optimal VIM-DBS contacts align along the cerebellothalamic pathway rather than to an anatomical sweet spot. Accumulating evidence from the present study and recent publications.

S4 Correlation maps from different VIM-DBS cohorts demonstrate a similar optimal connectivity profile

S5 Mean active contact standard deviation across VIM-DBS cohorts reveals distinct locations within the motor thalamus

S6 Replication of the main analysis including the Florida VIM-DBS cohort

S7 *Multimodally-informed convergent tremor map without including the atrophy map* anticipates clinical outcomes in out-of-sample GPi cohort

S8 Multimodally informed convergent tremor map estimates clinical improvement in tremor subscores

S9 Comparison of tremor subtypes

S10 Replication of main results using a disease specific connectome derived from the Parkinson's Progression Markers Initiative (PPMI) instead of the normative connectome derived from healthy subjects

S11 Methods overview of DBS network mapping

Supplementary Analyses

S1 DBS Cohort analyses

Method description

S1 Acquisition of EMG-fMRI map

Discussion

S1 Therapeutic effects of GPi-DBS in Parkinsonian tremor (including Fig. S12)

Tables

S1 Summary of clinical patient characteristics within each discovery cohort.

S2 Lesion network mapping (LNM) studies in motor symptoms: Summary of studies demonstrating how lesions in different brain regions can map onto common brain circuits.

Supplementary references

Figures

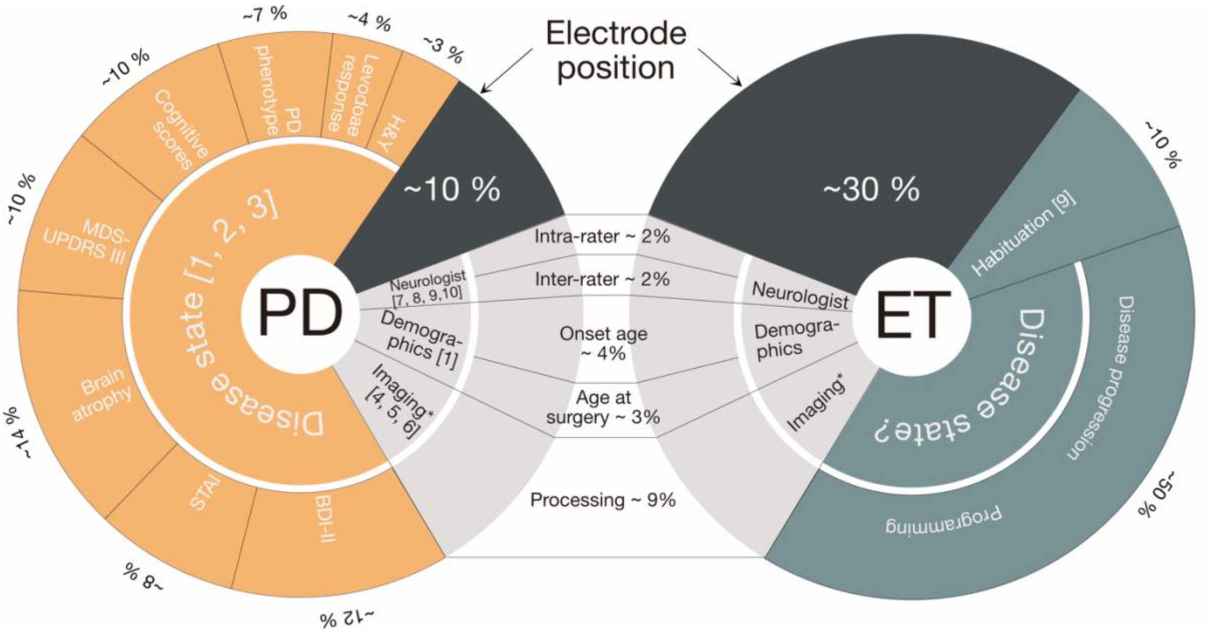

Fig. S1. Modeling considerations. The pie charts depict factors influencing patient outcomes following DBS, as determined through literature review. For Parkinson's disease (PD), more studies have investigated determinants of DBS outcomes compared to other conditions. Key considerations include how much variance DBS modeling can realistically explain. Clinical improvements depend on multiple factors beyond electrode placement and stimulation volumes, such as disease subtype, age, sex, levodopa response, disease duration, and comorbidities, which collectively account for approximately 50% of the variance in DBS response. Measurement noise, including inter- and intra-rater reliability of UPDRS-III scores, contributes another ~20%. Imaging resolution, electrode placement inaccuracies, and modeling limitations add ~10% variance in PD and ~30% in essential tremor (ET). Habituation effects are present particularly in VIM-DBS for ET. Further factors remain uncertain but may further influence outcomes. Despite these challenges, such models remain clinically valuable. Electrode placement and stimulation settings are modifiable, unlike immutable patient factors such as age or disease type. Consequently, identifying an optimal target that explains even ~10% of variance represents a meaningful advancement in the field [1–10].

*It is uncertain how much variance in explained clinical outcomes imaging reconstruction errors will have since no study deliberately tested for this. However, multiple reports speak of reconstruction errors of at least 1 mm displacement [4–6]. The only study that we are aware of which tested for artificially introduced spatial uncertainty imposed a jitter of N=258 electrode placements according to a 3D Gaussian distribution of 2 mm full width half maximum. In this report, the standard error interval in outcome predictions for iteratively jittered group analyses ranged from ~60 % and ~70 % explained variance, i.e. a standard error of around 10%.

Abbreviations: BDI-II: Beck Depression Inventory; DBS, deep brain stimulation; ET, Essential tremor; H&Y: Hoehn and Yahr Scale; MDS-UPDRS-III, Movement Disorder Society Unified Parkinson’s Disease Rating Scale – Part III; PD, Parkinson’s disease; STAI: State Trait Anxiety Inventory.

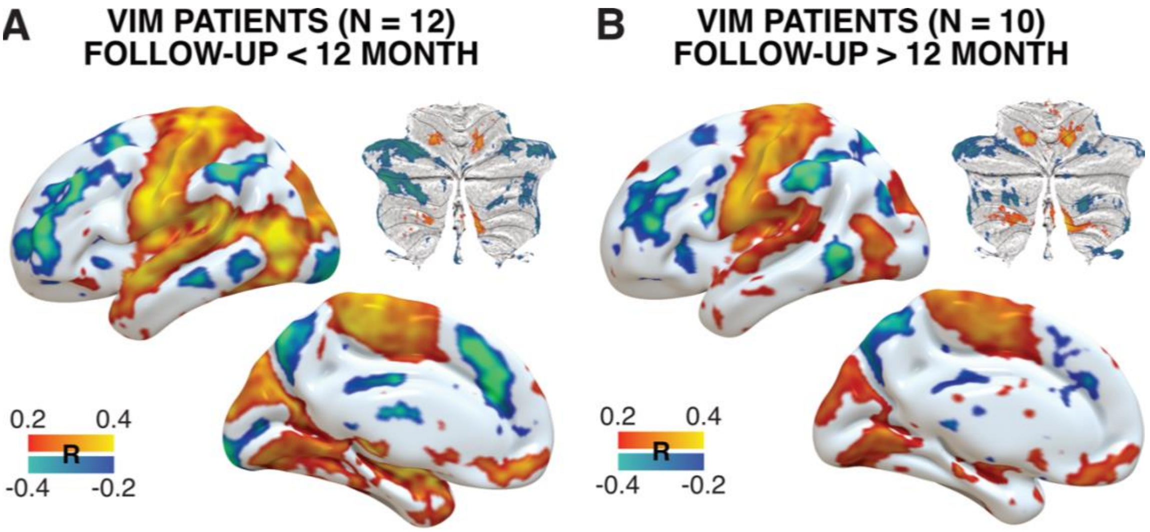

Fig. S2. Correlation maps for VIM-DBS patients based on follow-up duration. A: Correlation map for VIM-DBS patients with follow-up durations less than 12 months (mean follow-up: 4.42 ± 2.26 months). **B**: Correlation map for VIM-DBS patients with follow-up durations greater than 12 months (mean follow-up: 21.1 ± 13.86 months). Both maps still highlighted the same general profile with positive connectivity to motor strip and motor cerebellum. *Abbreviations:* DBS, deep brain stimulation; STN, subthalamic nucleus; VIM, ventral intermediate nucleus.

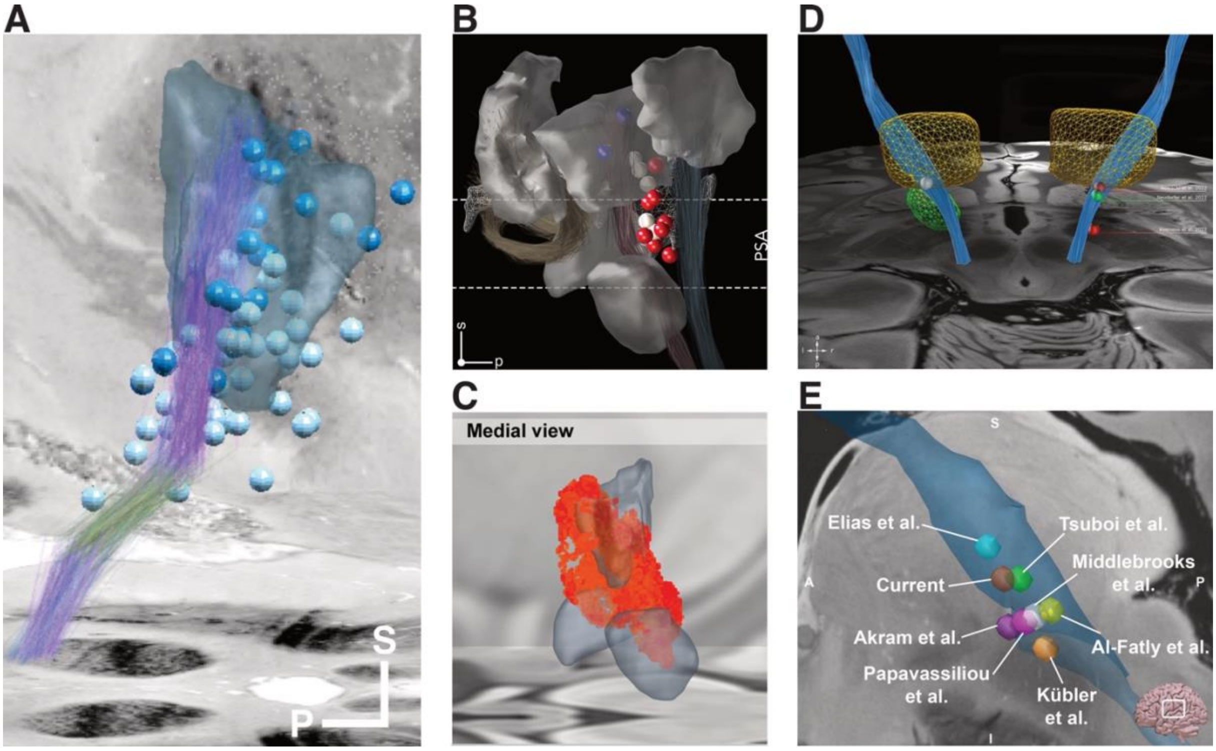

Fig. S3. Optimal VIM-DBS contacts align along the cerebellothalamic pathway rather than to an anatomical sweet spot. Accumulating evidence from the present study and recent publications. **A:** Active contacts of all VIM-DBS electrodes from the present study, including those from the Berlin cohort (light blue) and the validation Florida cohort (dark blue), aligned with the trajectory of the cerebellothalamic pathway / dentatorubrothalamic tract (DRT, purple and green). DRT fibers are shown as defined in the Basal Ganglia Pathway Atlas [11]. **B:** Medial view of the thalamus and posterior subthalamic area (PSA), showing previously reported target coordinates associated with symptom improvement. Sites linked to better clinical outcomes are closely related to the cerebellothalamic outflow tract (dark red) and the zona incerta (white mesh) at the level of the PSA. Panel adapted, with permission, from [12]. **C:** Medial view showing an excellent responder cluster from the largest meta-analysis to date in VIM-DBS for tremor, as recently identified. Panel adapted, with permission, from [13]. **D:** The white sphere represents the classic VIM target at the ventral border of the VIM nucleus (left side of the brain in posterior view), while recently recommended targets from the literature are shown on the right side of the brain in posterior view. The VIM nucleus is shown in yellow, and the DRT is displayed in blue. Panel adapted, with permission, from [14]. **E:** The dentatothalamic tract is shown in blue, alongside recently published sweet spots for optimal tremor control. Panel adapted, with permission, from [15]. *Abbreviations:* DBS, deep brain stimulation; PSA, posterior subthalamic area; VIM, ventral intermediate nucleus.

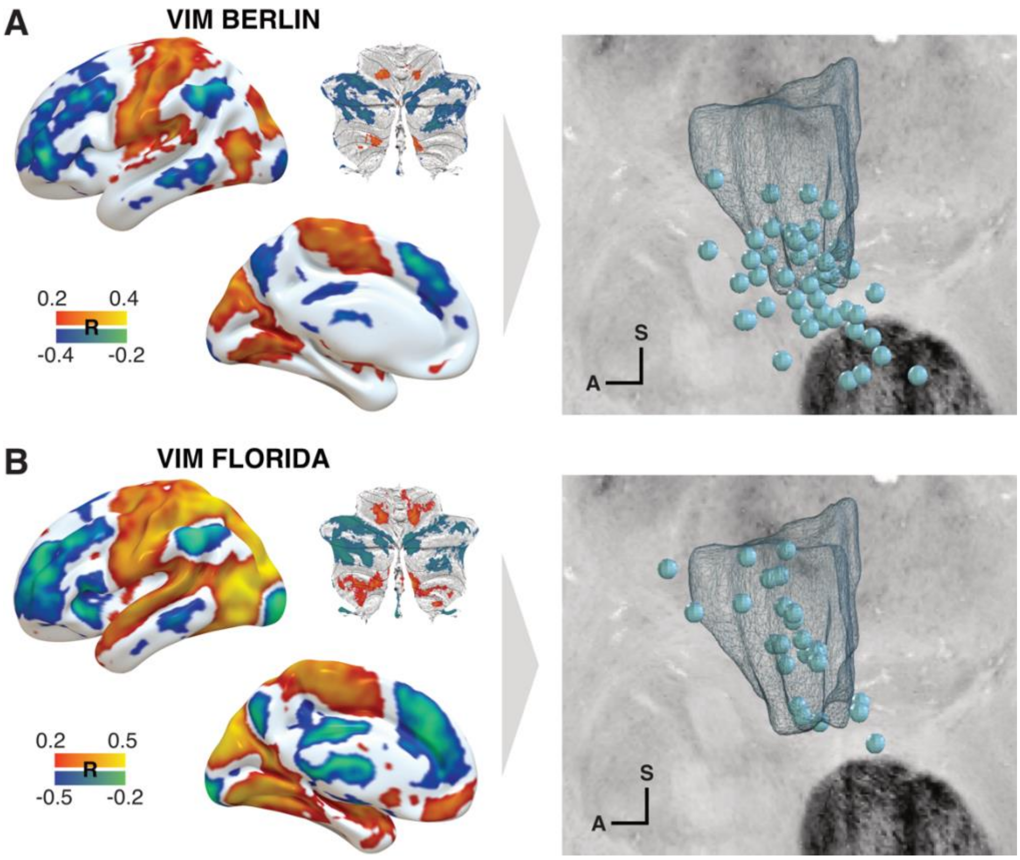

Fig. S4. Correlation maps from different VIM-DBS cohorts demonstrate a similar optimal connectivity profile. A: Correlation map (R-map) from the VIM-DBS cohort in Berlin (left) compared to the R-map from the Florida cohort (right) shows a similar optimal connectivity profile, despite variations in surgical approaches. **B:** Active contacts (dots) from the left hemisphere illustrate these differences, viewed from the left side. The BigBrain Atlas is used as the anatomical backdrop [16]. *Abbreviations:* DBS, deep brain stimulation; VIM, ventral intermediate nucleus.

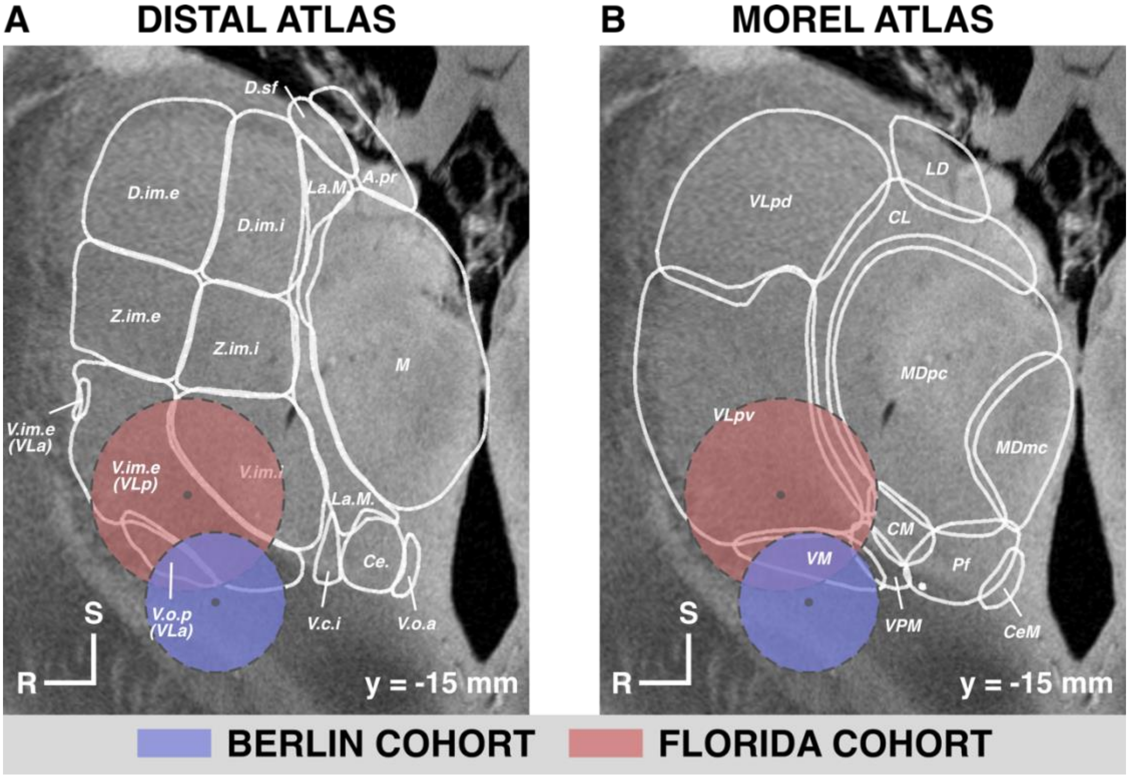

Fig. S5. Mean active contact standard deviation across VIM-DBS cohorts reveals distinct locations within the motor thalamus. The average active contacts (grey circle) and standard deviations (dashed circle line) are shown in light blue (Berlin) and light red (Florida). **A:** Outlines from DISTAL atlas [17]. *Abbreviations* (Hassler / Schaltenbrand & Wahren nomenclature): A.pr, Ncl. anterior principalis; Ce., Central nucleus; D.im.e, Dorsal intermediate nucleus, external part; D.im.i., Dorsal intermediate nucleus, internal part; D.sf, Dorsal superficial nucleus; La.M., Internal medullary lamina; M, Medial nucleus; V.c.i, Ventral caudal nucleus, internal part; V.im.i, Ventral intermediate nucleus, internal part; V.im.e, Ventral intermediate nucleus, external part; VL, Ventral lateral nucleus; V.o.a, Ventral oral anterior nucleus; V.o.p, Ventral oral posterior nucleus (VLa, ventral lateral anterior nucleus); Z.im.e, Ncl. zentro-lateralis intermedius, external part; Z.im.i, Ncl. zentro-lateralis intermedius, internal part. The 7 Tesla MRI of the ex vivo human brain is used as the anatomical backdrop [18]. **B:** Outlines from Morel atlas [19]. *Abbreviations* (nomenclature by Hirai and Jones and adopted by Morel et al. [19]): CeM, Central medial nucleus; CL, Central lateral nucleus; CM, Centromedian nucleus; LD, Lateral dorsal nucleus; MDmc, Mediodorsal nucleus, magnocellular part; MDpc, Mediodorsal nucleus, parvocellular part; Pf, Parafascicular nucleus; VLpd, Ventral lateral nucleus, posterior dorsal part; VLpv, Ventral lateral nucleus, posterior ventral part; VM, Ventral medial nucleus; VPM, Ventral posteromedial nucleus.

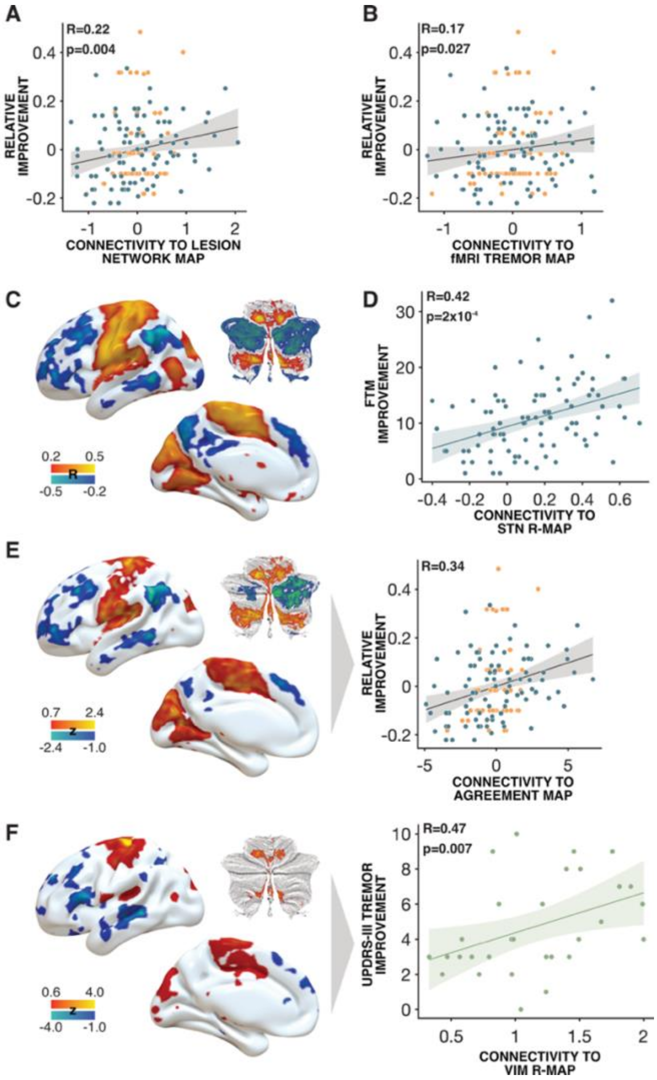

Fig. S6. Replication of the main analysis including the Florida VIM-DBS cohort. This analysis included N = 36 VIM-DBS patients from Berlin, Germany (bilateral DBS), N = 19 VIM-DBS patients from Florida, USA (unilateral DBS), and all STN-DBS patients from the main analysis. VIM patients are represented in blue, STN patients in orange. As a limitation, in the Florida cohort, only FTM sum scores were available, whereas the main analysis used the sum of specific FTM tremor score items to best match MDS-UPDRS-III tremor scores. **A:** Positive correlation between relative clinical improvement following DBS and each electrode's connectivity to the lesion network map correlation (rho(154) = 0.22, p = 0.004, 95% CI [0.07, 0.37]) and the fMRI-derived map (rho(154) = 0.17, p = 0.027, 95% CI [0.02, 0.32]) (original analyses in figures 1 and 2) **(B)**. **C:** Connectivity correlation map (R-map) derived from the full VIM-DBS cohort, including patients from both Berlin and Florida, as used in the replication analysis (original figure 3). This map represents the optimal connectivity profile associated with clinical improvement across the expanded VIM-DBS dataset. **D:** VIM-DBS electrodes' VTA connectivity to the STN cohort's optimal connectivity profile (R-map) correlated with clinical improvement (rho(89) = 0.42, p = $2x10^{-4}$, 95% CI [0.23, 0.58]) (original figure 3). **E:** Agreement map (left) combining STN and VIM cohorts when including both Berlin and Florida cohorts. Right panel: positive correlation between DBS site connectivity to the agreement map and clinical improvement (original figure 4). **F:** Multimodally-informed convergent tremor map, created by overlaying the lesion-derived map, EMG-fMRI-derived map, VIM-STN agreement map when including both Berlin and Florida cohorts, and an ET-specific atrophy map, with z-scores visualized. In an out-of-sample cohort of 31 PD hemispheres with GPi-DBS, connectivity to this map correlated with MDS-UPDRS-III tremor improvement (rho(29) = 0.47, p = 0.007, 95% CI [0.14, 0.71]) (original figure 5). A cohort regressor was applied in all analyses to account for potential confounding effects related to differences between patient groups. The shaded area indicates the 95% confidence interval. *Abbreviations:* DBS, deep brain stimulation; FTM, Fahn-Tolosa-Marin Clinical Rating Scale for Tremor; GPi, globus pallidus internus; UPDRS-III, Unified Parkinson's Disease Rating Scale – Part III; PD, Parkinson's disease; STN, subthalamic nucleus; VIM, ventral intermediate nucleus; VTA, volume of activated tissue.

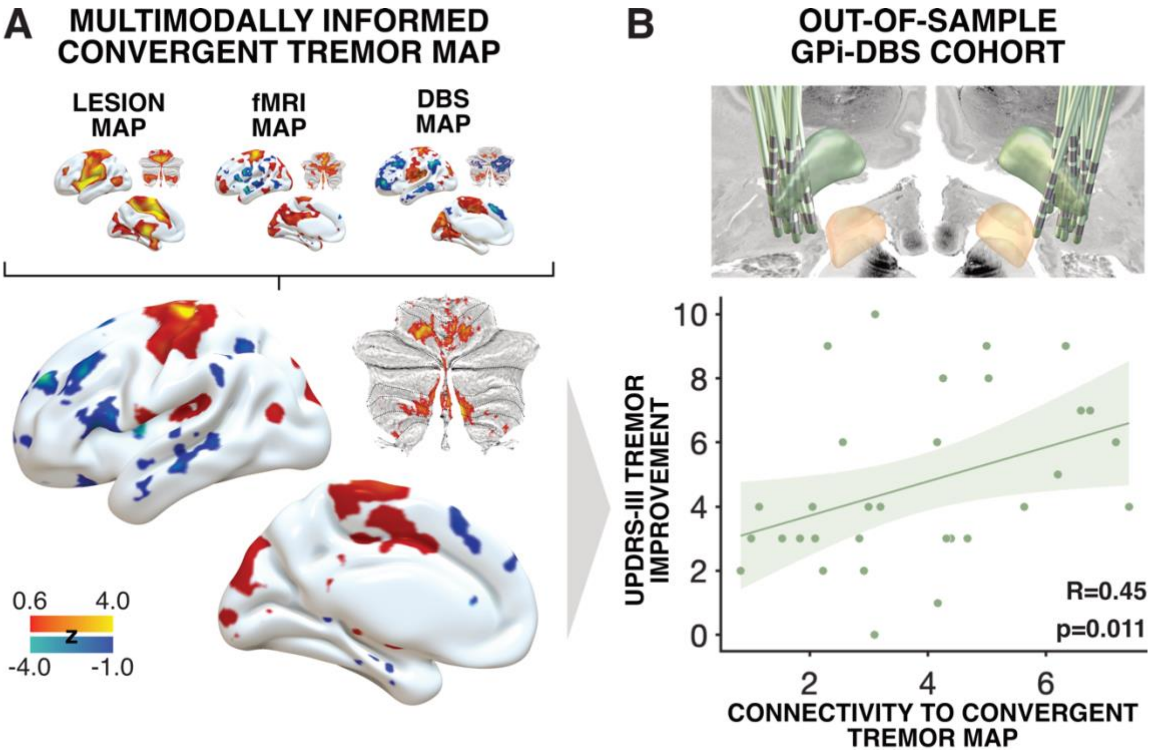

Fig. S7. ***Multimodally-informed convergent tremor map without including the atrophy map*** **anticipates clinical outcomes in out-of-sample GPi cohort. A:** The lesion-derived map, EMG-fMRI-derived map and the VIM-STN (agreement) map were superimposed to create the displayed *multimodally-informed convergent tremor map*, with z-scores visualized. **B:** In an out-of-sample cohort of 31 analyzed hemispheres from PD patients with GPi-DBS, lead localizations were conducted using Lead-DBS (localizations shown in the top right image) [20]. Correlation between connectivity of DBS sites to the convergent tremor map and MDS-UPDRS-III tremor improvements rho(29) = 0.45, p = 0.011, 95% CI [0.12, 0.70]. The shaded area indicates the 95% confidence interval. *Abbreviations:* DBS, deep brain stimulation; GPi, globus pallidus internus; UPDRS-III, Unified Parkinson's Disease Rating Scale – Part III; PD, Parkinson's disease.

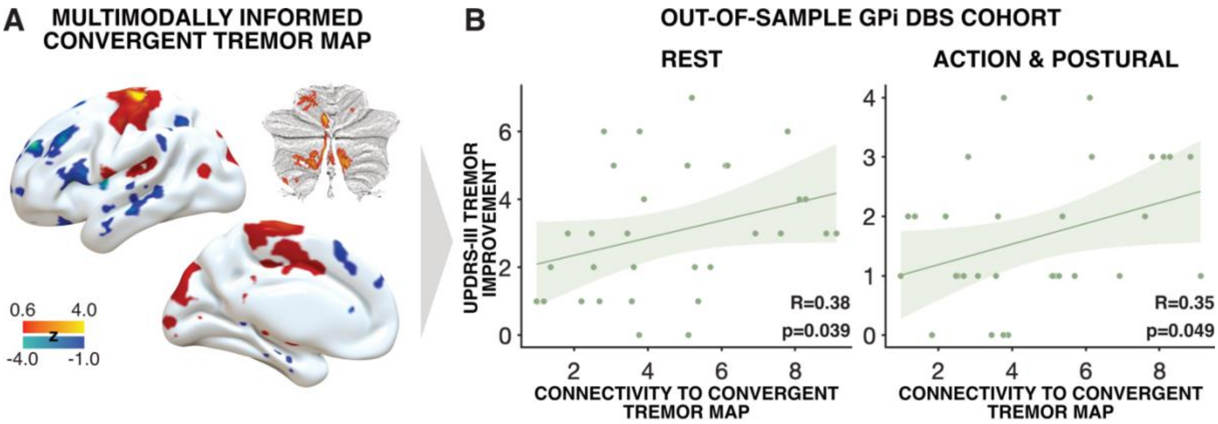

Fig. S8. Multimodally informed convergent tremor map estimates clinical improvement in tremor subscores. A: Multimodally-informed convergent tremor map, as shown in Fig. 5. **B:** Out-of-sample analysis of GPi connectivity to the convergent tremor map, with tremor improvement assessed using subscores for rest tremor and the combined action/postural tremor score. Connectivity to the convergent tremor map significantly correlated with improvement in both rest (rho(29) = 0.38, p = 0.039, 95% CI [0.02, 0.64]) and action/postural (rho(29) = 0.35, p = 0.049, 95% CI [0.00, 0.62]) tremor subscores. The shaded area indicates the 95% confidence interval. *Abbreviations:* DBS, deep brain stimulation; GPi, globus pallidus internus; UPDRS-III, Unified Parkinson's Disease Rating Scale – Part III.

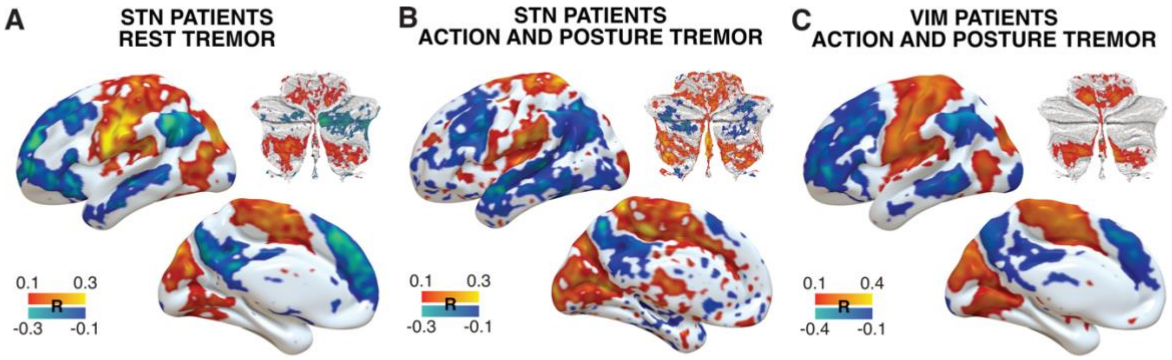

Fig. S9. Comparison of tremor subtypes. Correlation maps show networks associated with optimal clinical response to DBS, demonstrating similarities across stimulation targets, disease types, and tremor subtypes (rest, action, and posture tremor). **A**: Optimal connectivity for rest tremor in STN patients. **B**: Optimal connectivity for action and posture tremor in STN patients. **C**: Optimal connectivity for action and posture tremor in VIM patients. The maps highlight overlapping areas of connectivity associated with effective tremor suppression, suggesting a shared therapeutic network underlying tremor relief irrespective of these variables. *Abbreviations:* DBS, deep brain stimulation; STN, subthalamic nucleus; VIM, ventral intermediate nucleus.

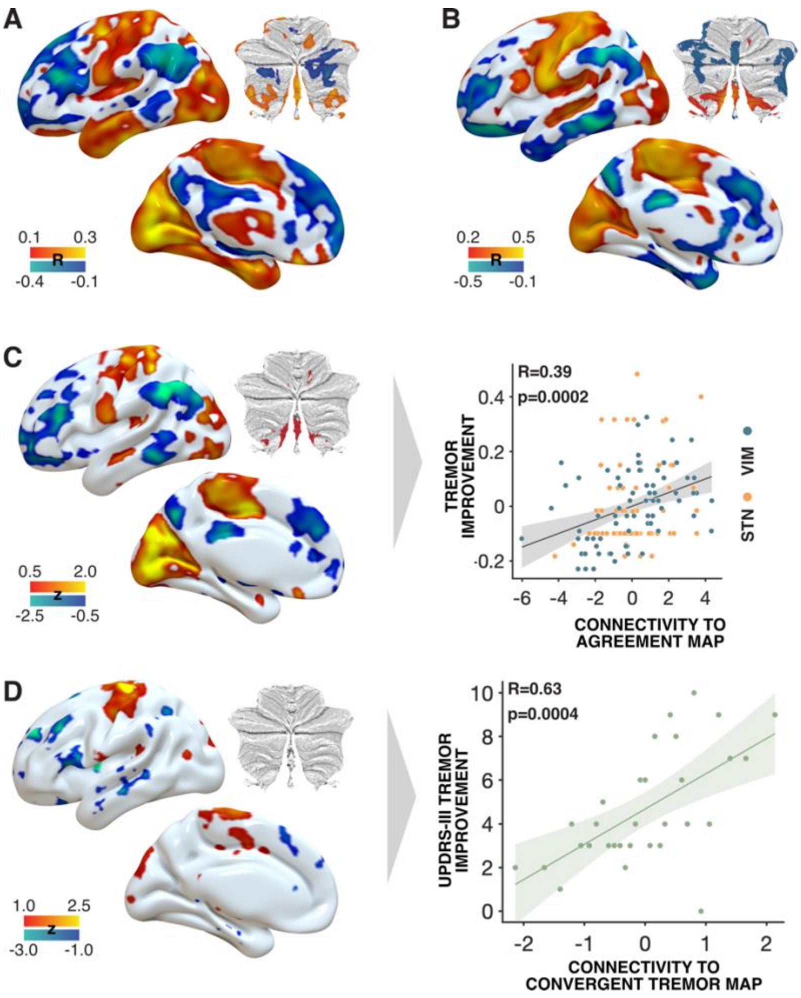

Fig. S10. Replication of main results using a disease specific connectome derived from the Parkinson's Progression Markers Initiative (PPMI) instead of the normative connectome derived from healthy subjects. A & B: Correlation maps representing optimal clinical improvement for STN-DBS patients and VIM-DBS patients, respectively (compare fig. 3). **C**: Agreement map consisting of STN-DBS- and VIM-DBS-correlation maps anticipates clinical improvement in VIM- and STN-DBS-patients (rho(135) = 0.39, p = 0.0002, 95% CI [0.24, 0.53]) (compare fig. 4). **D:** Multimodally-informed convergent tremor map consisting of lesion-derived fMRI-map, EMG-fMRI-derived map, VIM-STN (agreement) map and ET-specific atrophy-map anticipates clinical outcomes in out-of-sample GPi cohort (rho(29) = 0.63, p = 0.0004, 95% CI [0.36, 0.81]) (compare fig. 5). The shaded area indicates the 95% confidence interval. *Abbreviations:* DBS, deep brain stimulation; ET, essential tremor; GPi, globus pallidus internus; UPDRS-III, Unified Parkinson's Disease Rating Scale – Part III; PD, Parkinson's disease; STN, subthalamic nucleus; VIM, ventral intermediate nucleus.

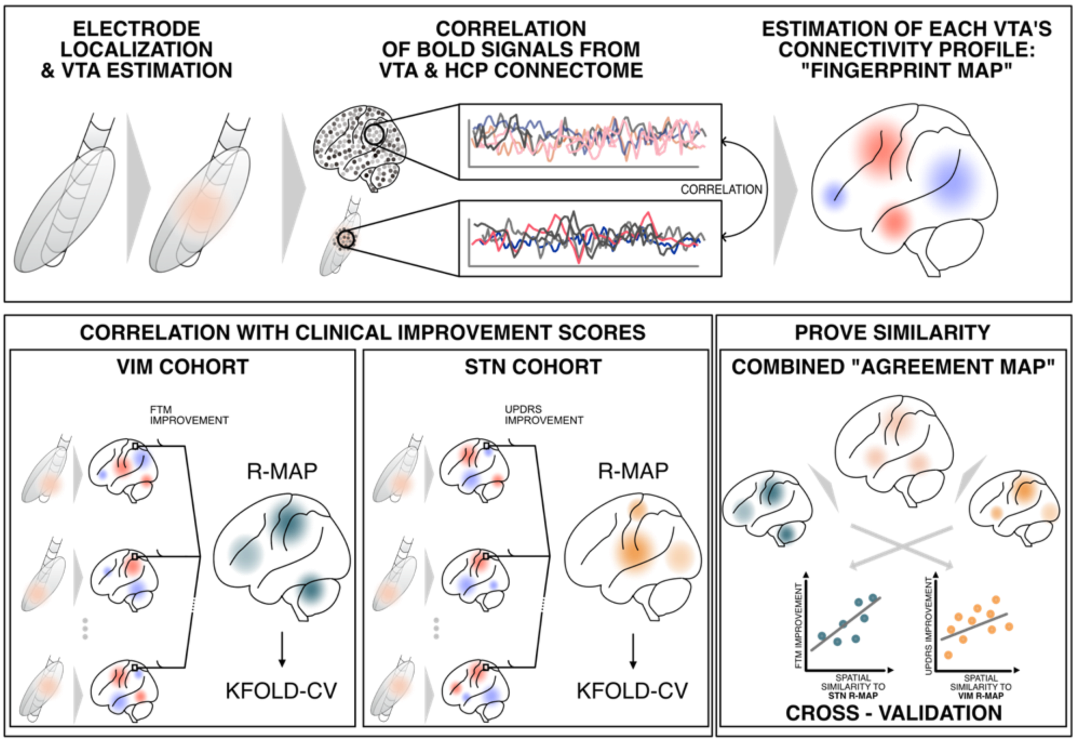

Fig. S11. Methods overview of DBS network mapping. DBS electrodes in all patients were localized using Lead-DBS software [20]. Seeding from voxels contained in the volume of activated tissue around each electrode, a functional connectivity profile was calculated, followed by voxel-wise correlation of clinical improvements with connectivity values. Across patients, connectivity values were correlated with tremor improvements, resulting in an *R-map* model of connectivity profile associated with maximal tremor response. R-maps of both cohorts were compared and combined to a map showing the agreement between *R-maps* of both cohorts. Cohorts were cross-validated by building a similarity index between the *R-map* of one cohort and the individual connectivity profile of each functional connectivity profile of the other cohorts' patients. In a final step, these were correlated with clinical improvement to test association between similarity in connectivity and clinical improvement. *Abbreviations:* CV, cross - validation; DBS, deep brain stimulation; FTM, Fahn-Tolosa-Marin Clinical Rating Scale for Tremor; HCP, Human Connectome Project; UPDRS-III, Unified Parkinson's Disease Rating Scale – Part III; STN, subthalamic nucleus; VIM, ventral intermediate nucleus; VTA, volume of activated tissue.

Supplementary Analyses

S1: DBS Cohort analyses

For the ET cohort, a generalized linear model (GLM) incorporating connectivity to the optimal model correlation map, baseline scores, patient age, and sex explained a significant proportion of the variance in clinical outcomes ($R^2 = 0.35$, $F = 35.8$, $p < 0.001$). Significant predictors included connectivity values ($t = 3.46$, $p < 0.001$) and baseline scores ($t = 10.05$, $p < 0.001$), whereas age ($p = 0.18$) and sex ($p = 0.64$) were not significant. Similarly, for the PD cohort, a GLM incorporating connectivity to the optimal model correlation map, baseline scores, patient age, and sex explained a substantial amount of variance in clinical outcomes ($R^2 = 0.42$, $F = 43.9$, $p < 0.001$). Significant predictors in this cohort were connectivity values ($t = 2.94$, $p = 0.005$) and baseline scores ($t = 11.13$, $p < 0.001$), while age ($p = 0.12$) and sex ($p = 0.08$) did not reach statistical significance. Notably, none of these variables can be influenced through medical practice, except for electrode placement and stimulation settings. This renders the model estimates, derived from these modifiable factors, a critical anchor point with the potential to improve patient care (also see Fig. S1).

To assess whether the duration of follow-up in VIM-DBS patients influenced the group map, we divided the cohort into two subgroups: patients with follow-up periods longer than 12 months (N = 10; >12 months) and those with shorter follow-up periods (N = 12; <12 months). Correlation maps of optimal clinical response still highlighted the same general profile with positive connectivity to motor strip and motor cerebellum (Fig. S2).

Since surgical approaches for DBS in ET may vary between centers, the optimal connectivity profile from an additional cohort from the Mayo Clinic Florida, Jacksonville, Florida [21] was compared to the VIM-DBS cohort analyzed in this study from Berlin and demonstrated similar regions involved in the optimal connectivity profile (see Figs. S4 and S5).

Supplementary method description

S1: Acquisition of EMG-fMRI map

The cohort consisted of 22 tremor-dominant PD patients who all had resting tremor during scanning [22]. The following steps were performed that led to the “fMRI tremor map” (Fig. 2):

Muscle activity of the most affected forearm (wrist flexors and extensors) was measured using MR-compatible EMG (Brain Products; sampling frequency 5000 Hz) during fMRI scanning in all 22 patients. BrainVision Analyzer 2.0 (Brain Products) was used for preprocessing the EMG data. Then we: (1) used MR artifact correction [23], (2) down-sampled to 1000 Hz, (3) filtered with a 20-200 Hz band-pass filter to remove movement artifacts, and (4) rectified the signal to enhance the information on EMG burst-frequency (tremor) of the signal, thereby also recovering the low- frequency EMG content [24]. Next, EMG data were analyzed using FieldTrip [25]. Specifically, we calculated the time-frequency representations (TFRs) between 1 and 20 Hz in steps of 0.1 s using a 2 s Hanning taper, which resulted in a 0.5 Hz resolution. By averaging over all time points, we obtained an average power spectrum across segments. For each patient, we picked the TFR of the corresponding tremor frequency (i.e., the peak in the power spectrum between 4 and 6 Hz; mean: 4.6 ± 0.1 Hz), resulting in patient-specific regressors describing fluctuations in tremor amplitude (EMG-AMP). To remove outliers, we calculated the logarithmic values of the EMG-AMP and z-normalized the data within subjects. Finally, the EMG-AMP regressor was convolved with the hemodynamic response function, and the resulting regressor was added to our first-level model in SPM, next to several regressors of no interest: two regressors describing the signal intensity averaged on each scan over the segmented gray matter (i.e., global signal, to correct for head motion [26]) and over a blank portion of the MR images (out-of-brain signal) and 36 regressors describing head motion. Regressors describing head motion were based on linear, quadratic, and cubic effects of the six movement parameters belonging to each volume, as well as the first and second derivative of each of those regressors (to control for spin-history effects [27]).

The “fMRI tremor map” (Fig. 2) are voxels where BOLD activity is significantly associated with fluctuations in tremor power during scanning, averaged across 22 tremor-dominant PD patients. This approach has been done and replicated in several studies, all showing the same cerebello-thalamo-cortical circuit [28–31].

Supplementary discussion

S1: Therapeutic effects of GPi-DBS in Parkinsonian tremor

A key question of our results is how GPi-DBS (as used to treat PD tremor) yields effective outcomes despite being outside the cerebello-thalamo-cortical circuitry that we identify as the main surrogate of tremor effects based on multi-modal data in the present manuscript. The following thoughts may help conceptualize this seeming paradox:

1. GPi as a "switch" for tremor episodes

Recent models suggest that the GPi may act as a trigger for Parkinsonian tremor episodes, while the cerebello-thalamo-cortical circuit serves as a “dimmer” controlling tremor amplitude, as already discussed in the main text of this manuscript (see discussion and [22]). This aligns with previous studies showing that STN stimulation, which also influences GPi, can effectively reduce tremor. The ability of GPi-DBS to suppress tremor may thus stem from its role in modulating the entire tremor-generating network rather than acting at a single focal target. While an empirical analysis demonstrating this relationship is not possible using methods of the present manuscript, we aimed at estimating the influence of voxels within the pallidal region onto the tremor network by calculating each voxel’s normative connectivity profile and spatially comparing its similarity to the tremor network. This led to peaks in voxels which had a connectivity profile most closely matching the profile of the convergent tremor network identified here. This analysis showed that the voxels with the strongest similarity precisely matched the posterolateral GPi region (matching the DBS target used in PD; figure S12).

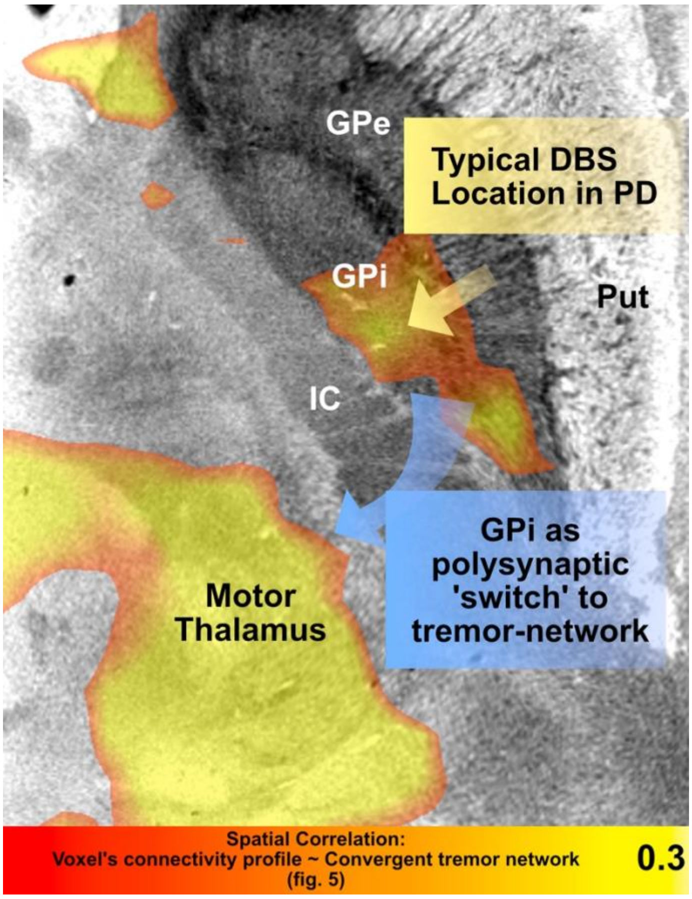

Fig. S12. Spatial correlation between connectivity profiles of all pallidal voxels to the multimodally informed convergent tremor network. For each voxel in the subcortical field of view, functional connectivity was seeded and averaged across 1,000 rs-fMRI scans in the normative HCP connectome. These profiles were then compared to the convergent tremor network (fig. 5) using spatial correlations. High correlations represent voxels whose connectivity profile matches the spatial properties of the multimodal tremor network more precisely than others. Within the pallidum, this analysis peaked within its dorsolateral sensorimotor region, precisely matching the typical location of DBS electrodes implanted to treat PD symptoms (including tremor). While indirect, this analysis may

support the notion that GPi may function as a polysynaptic 'switch' to modulate the tremor network as originally hypothesized in [22]. *Abbreviations:* DBS, deep brain stimulation; GPe, globus pallidus externus; GPi, globus pallidus internus; IC, internal capsule; PD, Parkinson's disease; Put, Putamen.

2. Network interactions between the basal ganglia and cerebello-thalamo-cortical circuit

Parkinsonian tremor differs fundamentally from other forms of tremor (e.g., essential tremor) in that it originates from pathological oscillations within the basal ganglia, which secondarily modulate the cerebello-thalamo-cortical circuit. This suggests that targeting GPi, an upstream nucleus within the basal ganglia-thalamo-cortical loop, can interrupt aberrant tremor activity before it propagates downstream. This is consistent with prior work showing that GPi acts as a key relay in the tremor circuitry, influencing both the basal ganglia and cerebello-thalamo-cortical pathways [32].

3. Tremor cells in the GPi and their role in parkinsonian tremor

Electrophysiological recordings have identified tremor cells within the lower portion of GPi, with activity that is highly correlated with tremor frequency in Parkinson's disease [32]. This suggests that GPi may play a direct role in tremor generation in PD, in contrast to essential tremor, which is primarily driven by cerebellothalamic dysfunction. Furthermore, cerebral activity related to Parkinson's tremor has been shown to first arise in the GPi and then propagate to the cerebello-thalamo-cortical circuit, suggesting that GPi stimulation might disrupt this pathological loop at its origin [22].

4. Influence of dopamine on GPi and tremor suppression

Dopamine is known to reduce tremor-related activity in both thalamus and GPi. In Parkinson's disease, dopaminergic depletion leads to pathological synchrony within the GPi, contributing to the emergence of resting tremor [28]. The fact that GPi-DBS improves tremor suggests that it may restore normal basal ganglia output and thereby indirectly modulate downstream (cerebellothalamic) tremor circuits.

5. The role of diaschisis and lesion network mapping (LNM) in understanding GPi effects

An additional parallel to understanding remote network effects of specific stimulation sites may be derived from results in the lesion network mapping (LNM) literature. Traditional lesion studies have demonstrated that symptoms such as tremor [33], hemichorea [34], or parkinsonism [35] do not always localize to a single anatomical region but rather to a distributed functional network. Even when lesion locations vary, they often map onto the same functionally connected network via the phenomenon of diaschisis, the remote impact of a lesion on connected brain regions [36].

In our case, GPi-DBS may leverage similar mechanisms, exerting network-level effects despite being anatomically distinct from the cerebello-thalamo-cortical tremor circuit. This aligns with prior LNM studies showing that lesions in different brain locations can converge onto a shared symptom-specific network (for examples, see table S2). If a lesion anywhere within a tremor network may cause tremor, then neuromodulation of a functionally connected region, such as the GPi, may similarly alleviate tremor, even if it is not the direct site of pathophysiology.

Supplementary table 1 (table S1): Summary of clinical patient characteristics within each discovery cohort.

Cohort (sorted after DBS target)	VIM		STN			GPi	
Underlying disease	ET	ET	PD	PD	PD	PD	PD
Surgical DBS center	Charité - Universitätsmedizin Berlin	Mayo Clinic Florida, Jacksonville	Charité - Universitätsmedizin Berlin	University Hospital Würzburg	Academic Medical Center, Amsterdam	Academic Medical Center, Amsterdam	Ruijin Hospital Shanghai
N (female)	36 (14)	19 (8)	18 (8)	13 (6)	16 (2)	11 (4)	11 (6)
Main clinical outcome assessment	FTM (Hemi)	FTM (Bilat.)	UPDRS-III	UPDRS-III	UPDRS-III	UPDRS-III	UPDRS-III
Score at baseline (mean ± SD)	13.85 (± 4.47)	41.47 (± 10.86)	4.50 (± 1.69)	4.44 (± 1.46)	5.09 (± 2.52)	6.25 (±2.49)	4.07 (±1.03)
Score at time of follow-up under stimulation ON condition (mean ± SD)	4.57 (± 3.02)	20.95 (± 8.78)	0.90 (± 1.44)	0.69 (± 1.01)	0.17 (± 0.58)	0 (±0)	1.07 (±1.33)
Relative improvement (mean ± SD)	0.64 (± 0.23)	0.50 (± 0.18)	0.85 (± 0.23)	0.86 (± 0.21)	0.98 (± 0.06)	1 (±1)	0.74 (±0.32)
Absolute improvement (mean ± SD)	9.28 (± 4.78)	20.52 (± 8.16)	3.6 (± 1.14)	3.75 (± 1.57)	4.91 (± 2.17)	6.25 (±2.49)	3.00 (±1.46)
Related citation	Al-Fatly et al., 2019	Neudorfer et al., 2022	Horn et al., 2017	Horn et al., 2017	Odekerken et al., 2013	Odekerken et al., 2013	Zhang et al., 2021

Notes: From the full cohort, only patients with a tremor score greater than two were included. For each hemisphere, the UPDRS-III assessment focused on kinetic tremor of the upper limb and rest tremor of both upper and lower limbs. In the FTM assessment, only limb tremor items for each hemisphere were included for the Berlin cohort analyzed in the main study. For the ET cohort from Florida, used to compare electrode placement sites, only total FTM scores were available. *Abbreviations:* DBS, deep brain stimulation; ET, Essential tremor; FTM, Fahn-Tolosa-Marin Clinical Rating Scale for Tremor; GPi, globus pallidus internus; PD, Parkinson’s disease; SD, standard deviation; STN, subthalamic nucleus; UPDRS-III, Unified Parkinson’s Disease Rating Scale – Part III; VIM, ventral intermediate nucleus.

Supplementary table 2 (table S2): Lesion network mapping (LNM) studies in motor symptoms: Summary of studies demonstrating how lesions in different brain regions can map onto common brain circuits.

Motor symptom	N	Description
Cervical dystonia [37]	25	Lesions inducing cervical dystonia showed positive connectivity to the cerebellum and negative connectivity to the somatosensory cortex.
Freezing of gait [38]	14	Anatomically heterogenic lesions showed in 13/14 cases network overlap in the dorsal medial cerebellum.
Hemichorea [34]	29	Anatomically heterogenic lesions showed in 90% of the cases network overlap in the posterolateral putamen.
Holmes' tremor [39]	26	Cases were connected to a common brain circuit with nodes in the red nucleus, thalamus, globus pallidus, and cerebellum.
Parkinsonism [35]	29	Most sensitive and specific connectivity was to the claustrum. 31 % of lesions hit the substantia nigra.
Tics [40]	22	Lesions mapped to a common brain circuit involving insular cortices, cingulate gyrus, striatum, globus pallidus internus, thalami and cerebellum. Connectivity to the putamen was specific to tic-inducing lesions.

References

1. Cavallieri, F. *et al.* Predictors of Long-Term Outcome of Subthalamic Stimulation in Parkinson Disease. *Annals of Neurology* **89**, 587–597 (2021).
2. Muthuraman, M. *et al.* Effects of DBS in parkinsonian patients depend on the structural integrity of frontal cortex. *Sci Rep* **7**, 43571 (2017).
3. Soulas, T., Sultan, S., Gurruchaga, J.-M., Palfi, S. & Fénelon, G. Depression and Coping as Predictors of Change After Deep Brain Stimulation in Parkinson's Disease. *World Neurosurgery* **75**, 525–532 (2011).
4. Noecker, A. M. *et al.* StimVision v2: Examples and Applications in Subthalamic Deep Brain Stimulation for Parkinson's Disease. *Neuromodulation: Technology at the Neural Interface* **24**, 248–258 (2021).
5. Maks, C. B., Butson, C. R., Walter, B. L., Vitek, J. L. & McIntyre, C. C. Deep brain stimulation activation volumes and their association with neurophysiological mapping and therapeutic outcomes. *Journal of Neurology, Neurosurgery & Psychiatry* **80**, 659–666 (2009).
6. Rajamani, N. *et al.* Deep brain stimulation of symptom-specific networks in Parkinson's disease. *Nat Commun* **15**, 4662 (2024).
7. Bennett, D. A., Shannon, K. M., Beckett, L. A., Goetz, C. G. & Wilson, R. S. Metric properties of nurses' ratings of parkinsonian signs with a modified Unified Parkinson's Disease Rating Scale. *Neurology* **49**, 1580–1587 (1997).
8. Siderowf, A. *et al.* Test–Retest reliability of the Unified Parkinson's Disease Rating Scale in patients with early Parkinson's disease: Results from a multicenter clinical trial. *Movement Disorders* **17**, 758–763 (2002).
9. Richards, M., Marder, K., Cote, L. & Mayeux, R. Interrater reliability of the unified Parkinson's disease rating scale motor examination. *Movement Disorders* **9**, 89–91 (1994).
10. Metman, L. V. *et al.* Test–retest reliability of UPDRS-III, dyskinesia scales, and timed motor tests in patients with advanced Parkinson's disease: An argument against multiple baseline assessments. *Movement Disorders* **19**, 1079–1084 (2004).
11. Petersen, M. V. *et al.* Holographic Reconstruction of Axonal Pathways in the Human Brain. *Neuron* **104**, 1056-1064.e3 (2019).
12. Neudorfer, C. *et al.* The role of the motor thalamus in deep brain stimulation for essential tremor. *Neurotherapeutics* **21**, e00313 (2024).
13. Nowacki, A. *et al.* Probabilistic Mapping Reveals Optimal Stimulation Site in Essential Tremor. *Annals of Neurology* **91**, 602–612 (2022).
14. Fox, M. D. & Deuschl, G. Converging on a Neuromodulation Target for Tremor. *Annals of Neurology* **91**, 581–584 (2022).
15. Middlebrooks, E. H. *et al.* Connectivity correlates to predict essential tremor deep brain stimulation outcome: Evidence for a common treatment pathway. *NeuroImage: Clinical* **32**, 102846 (2021).
16. Amunts, K. *et al.* BigBrain: An Ultrahigh-Resolution 3D Human Brain Model. *Science* **340**, 1472–1475 (2013).
17. Ewert, S. *et al.* Toward defining deep brain stimulation targets in MNI space: A subcortical atlas based on multimodal MRI, histology and structural connectivity. *NeuroImage* **170**, 271–282 (2018).
18. Edlow, B. L. *et al.* 7 Tesla MRI of the ex vivo human brain at 100 micron resolution. *Sci Data* **6**, 244 (2019).
19. Morel, A. *Stereotactic Atlas of the Human Thalamus and Basal Ganglia*. (CRC Press, Boca Raton, 2007). doi:10.3109/9781420016796.
20. Neudorfer, C. *et al.* Lead-DBS v3.0: Mapping deep brain stimulation effects to local anatomy and global networks. *NeuroImage* **268**, 119862 (2023).
21. Neudorfer, C. *et al.* Personalizing Deep Brain Stimulation Using Advanced Imaging Sequences. *Annals of Neurology* **91**, 613–628 (2022).
22. Dirkx, M. F. *et al.* The Cerebral Network of Parkinson's Tremor: An Effective Connectivity fMRI Study. *J. Neurosci.* **36**, 5362–5372 (2016).
23. Allen, P. J., Josephs, O. & Turner, R. A Method for Removing Imaging Artifact from Continuous EEG Recorded during Functional MRI. *NeuroImage* **12**, 230–239 (2000).
24. Myers, L. J. *et al.* Rectification and non-linear pre-processing of EMG signals for cortico-muscular analysis. *Journal of Neuroscience Methods* **124**, 157–165 (2003).
25. Oostenveld, R., Fries, P., Maris, E. & Schoffelen, J.-M. FieldTrip: Open source software for advanced analysis of MEG, EEG, and invasive electrophysiological data. *Comput Intell Neurosci* **2011**, 156869 (2011).
26. Power, J. D. *et al.* Methods to detect, characterize, and remove motion artifact in resting state fMRI. *NeuroImage* **84**, 320–341 (2014).

27. Lund, T. E., Nørgaard, M. D., Rostrup, E., Rowe, J. B. & Paulson, O. B. Motion or activity: their role in intra- and inter-subject variation in fMRI. *NeuroImage* **26**, 960–964 (2005).
28. Helmich, R. C., Janssen, M. J. R., Oyen, W. J. G., Bloem, B. R. & Toni, I. Pallidal dysfunction drives a cerebellothalamic circuit into Parkinson tremor. *Ann Neurol.* **69**, 269–281 (2011).
29. Dirkx, M. F. *et al.* Dopamine controls Parkinson's tremor by inhibiting the cerebellar thalamus. *Brain* **140**, 721–734 (2017).
30. Dirkx, M. F. *et al.* Cerebral differences between dopamine-resistant and dopamine-responsive Parkinson's tremor. *Brain* **142**, 3144–3157 (2019).
31. Dirkx, M. F., Shine, J. M. & Helmich, R. C. Integrative Brain States Facilitate the Expression of Parkinson's Tremor. *Mov Disord* **38**, 1615–1624 (2023).
32. Deuschl, G. *et al.* The pathophysiology of parkinsonian tremor: a review. *J Neurol* **247**, V33–V48 (2000).
33. Joutsa, J. *et al.* Identifying therapeutic targets from spontaneous beneficial brain lesions. *Annals of Neurology* **84**, 153–157 (2018).
34. Laganiere, S., Boes, A. D. & Fox, M. D. Network localization of hemichorea-hemiballismus. *Neurology* **86**, 2187–2195 (2016).
35. Joutsa, J., Horn, A., Hsu, J. & Fox, M. D. Localizing parkinsonism based on focal brain lesions. *Brain* **141**, 2445–2456 (2018).
36. Joutsa, J., Corp, D. T. & Fox, M. D. Lesion network mapping for symptom localization: recent developments and future directions. *Current Opinion in Neurology* **35**, 453–459 (2022).
37. Corp, D. T. *et al.* Network localization of cervical dystonia based on causal brain lesions. *Brain* **142**, 1660–1674 (2019).
38. Fasano, A., Laganiere, S. E., Lam, S. & Fox, M. D. Lesions causing freezing of gait localize to a cerebellar functional network. *Annals of Neurology* **81**, 129–141 (2017).
39. Joutsa, J., Shih, L. C. & Fox, M. D. Mapping holmes tremor circuit using the human brain connectome. *Ann Neurol* **86**, 812–820 (2019).
40. Ganos, C. *et al.* A neural network for tics: insights from causal brain lesions and deep brain stimulation. *Brain* **145**, 4385–4397 (2022).
