# Supplementary material for: Convergent mapping of a tremor treatment network
Source: Nat Commun. 2025 May 22;16:4772. doi: 10.1038/s41467-025-60089-6 (PMC12098757; doi:10.1038/s41467-025-60089-6)
Supplement: Supplementary file 2 — Reporting Summary [file 41467_2025_60089_MOESM2_ESM.pdf]

Corresponding author(s): Lukas Goede

Last updated by author(s): Oct 15, 2024

Reporting Summary

Nature Portfolio wishes to improve the reproducibility of the work that we publish. This form provides structure for consistency and transparency in reporting. For further information on Nature Portfolio policies, see our Editorial Policies and the Editorial Policy Checklist.

Statistics

For all statistical analyses, confirm that the following items are present in the figure legend, table legend, main text, or Methods section.

n/a	Confirmed	
☐	☒	The exact sample size (*n*) for each experimental group/condition, given as a discrete number and unit of measurement
☐	☒	A statement on whether measurements were taken from distinct samples or whether the same sample was measured repeatedly
☐	☒	The statistical test(s) used AND whether they are one- or two-sided<br>*Only common tests should be described solely by name; describe more complex techniques in the Methods section.*
☐	☒	A description of all covariates tested
☐	☒	A description of any assumptions or corrections, such as tests of normality and adjustment for multiple comparisons
☐	☒	A full description of the statistical parameters including central tendency (e.g. means) or other basic estimates (e.g. regression coefficient) AND variation (e.g. standard deviation) or associated estimates of uncertainty (e.g. confidence intervals)
☐	☒	For null hypothesis testing, the test statistic (e.g. *F*, *t*, *r*) with confidence intervals, effect sizes, degrees of freedom and *P* value noted<br>*Give P values as exact values whenever suitable.*
☒	☐	For Bayesian analysis, information on the choice of priors and Markov chain Monte Carlo settings
☒	☐	For hierarchical and complex designs, identification of the appropriate level for tests and full reporting of outcomes
☐	☒	Estimates of effect sizes (e.g. Cohen's *d*, Pearson's *r*), indicating how they were calculated

Our web collection on statistics for biologists contains articles on many of the points above.

Software and code

Policy information about availability of computer code

Data collection	Microsoft Excel Version 16.89.1 & Matlab R2023a
Data analysis	MATLAB R2023a<br>Lead-DBS v2 (including tools: adapted algorithms from SPM12, Advanced Normalization Tools, PaCER, Simbio, Fieldtrip): https://github.com/netstim/leaddbs, https://github.com/netstim/SlicerNetstim<br>3D Slicer Version 5.0.3

For manuscripts utilizing custom algorithms or software that are central to the research but not yet described in published literature, software must be made available to editors and reviewers. We strongly encourage code deposition in a community repository (e.g. GitHub). See the Nature Portfolio guidelines for submitting code & software for further information.

Data

Policy information about availability of data

All manuscripts must include a data availability statement. This statement should provide the following information, where applicable:

- Accession codes, unique identifiers, or web links for publicly available datasets
- A description of any restrictions on data availability
- For clinical datasets or third party data, please ensure that the statement adheres to our policy

Due to privacy regulations concerning patient health information, patient-specific imaging data is not publicly available but can be obtained from the corresponding

author upon request. The connectivity data generated in this study are provided in the Source Data file. Atlases used for visualization are openly available within Lead-DBS software (www.lead-dbs.org).

Research involving human participants, their data, or biological material

Policy information about studies with human participants or human data. See also policy information about sex, gender (identity/presentation), and sexual orientation and race, ethnicity and racism.

Reporting on sex and gender	Our study did initially not include a sex- or gender-based analysis due to the lack of prior literature indicating differences in symptom networks and circuits between sexes or genders. Consequently, we pooled data across these groups and conducted our analysis on the combined dataset. During the review process, an additional analysis was performed, which found that sex did not account for a significant amount of variance in clinical outcomes.
Reporting on race, ethnicity, or other socially relevant groupings	Our study did not include race or ethnicity as variables in the analysis due to the absence of evidence suggesting significant differences in symptom networks or circuits between these groups in the context of our research.
Population characteristics	The supplementary table 1 provides information about the individual cohorts and citation of the original publication including more details about the specific cohort characteristics.
Recruitment	Patients were retrospectively analyzed based on previously published data summarized in the text and in table S1.
Ethics oversight	The study was conducted in accordance with the Declaration of Helsinki and approved by the institutional review board of Charité – Universitätsmedizin Berlin (master vote EA2/186/18).

Note that full information on the approval of the study protocol must also be provided in the manuscript.

Field-specific reporting

Please select the one below that is the best fit for your research. If you are not sure, read the appropriate sections before making your selection.

☒ Life sciences ☐ Behavioural & social sciences ☐ Ecological, evolutionary & environmental sciences

For a reference copy of the document with all sections, see nature.com/documents/nr-reporting-summary-flat.pdf

Life sciences study design

All studies must disclose on these points even when the disclosure is negative.

Sample size	Given the exploratory nature of our study, conducting an a priori power analysis was challenging. We based our expected effect size on Al-Fatly et al. (Brain, 2019), with an r = 0.36 for reported correlations. Considering the limitations in available sample sizes across DBS targets, we performed a "compromise" power analysis using G*Power Version 3.1.9.6 (Faul et al., 2007; 2009, Behav. Res. Methods) to estimate the power of our analysis. We assumed equal Type I (α) and Type II (β) error probabilities, with a ratio of 1. The power estimates were as follows: VIM cohort (n = 72 hemispheres) had a power of 0.93 (α/β error probability = 0.07), STN cohort (n = 65 hemispheres) had a power of 0.91 (α/β error probability = 0.09), and GPi cohort (n = 31 hemispheres) had a power of 0.80 (α/β error probability = 0.20). These power values suggest that the VIM and STN cohorts had strong statistical power to detect the expected correlations, while the GPi cohort demonstrated adequate power for reliable detection.
Data exclusions	Data from patients with less than 3 tremor points were excluded.
Replication	We assessed the reproducibility of our cohorts in explaining clinical improvements in hold-out data using a five-fold cross-validation design. Additionally, the final convergent tremor map was able to estimate clinical outcomes in an out-of-sample discovery cohort.
Randomization	Our study compared the optimal connectivity profiles associated with symptom improvement across different DBS cohorts. Group affiliation was determined by DBS target, and no randomization step was performed.
Blinding	Blinding was not applicable to the main analysis of our study, as it involved an analysis of existing datasets. To minimize the risk of observer bias, we assessed the explanatory value using a hold-out analysis with five-fold cross-validation. Additionally, we conducted permutation testing.

Reporting for specific materials, systems and methods

We require information from authors about some types of materials, experimental systems and methods used in many studies. Here, indicate whether each material, system or method listed is relevant to your study. If you are not sure if a list item applies to your research, read the appropriate section before selecting a response.

Materials & experimental systems

n/a	Involved in the study
☒	☐ Antibodies
☒	☐ Eukaryotic cell lines
☒	☐ Palaeontology and archaeology
☒	☐ Animals and other organisms
☒	☐ Clinical data
☒	☐ Dual use research of concern
☒	☐ Plants

Methods

n/a	Involved in the study
☒	☐ ChIP-seq
☒	☐ Flow cytometry
☐	☐ MRI-based neuroimaging

Plants

Seed stocks: n/a

Novel plant genotypes: n/a

Authentication: n/a

Magnetic resonance imaging

Experimental design

Design type: Retrospective analysis of structural cerebral MRI and / or CT combined with normative functional human connectome data.

Design specifications: Individualized structural MRI and/or CT of the head combined with normative structural human connectome data
Patient-specific structural MRI or CT scans of the head were used to localize stimulation sites (Neudorfer et al., 2023, Neuroimage).

Further, a normative connectome was created based on time series of resting state fMRI acquired in 1,087 healthy subjects from the Human Connectome Project (HCP; http://www.humanconnectomeproject.org) cohort. The data had been acquired using specialized magnetic resonance hardware 55 and are publicly available in 'minimally preprocessed' form in MNI space. Time series in four runs per subject acquired at a repetition rate of 720 ms were concatenated after mean-averaging each run, then correlation coefficients between each pair of 2 mm isotropic voxels in MNI space were calculated, leading to a 285,903 × 285,903 adjacency matrix per subject. Matrices were averaged across the 1,087 subjects to embody a normative 'group connectome' that was used to estimate connectivity profiles seeding from each DBS stimulation field, going forward.

Behavioral performance measures: Disease-specific outcome scores were used: the motor section of the Unified Parkinson's Disease Rating Scale (UPDRS-III) for Parkinson's disease and the Fahn-Tolosa-Marin Clinical Rating Scale for Tremor (FTM) for essential tremor patients.

Acquisition

Imaging type(s): Structural

Field strength: 1.5

Sequence & imaging parameters: Pre operative T1-weighted MRI, pre-operative T2-weighted MRI, Post-op CT and postop T1-weighted MRI

Area of acquisition: Whole brain

Diffusion MRI: ☐ Used ☒ Not used

Preprocessing

Preprocessing software: SPM, ANTs, LeadDBS v3, DSI-studio v3

Normalization: ANTs and SPM

Normalization template | ICBM 2009b Non linear asymmetric space

Noise and artifact removal | Biasfield correction, Brain shift correction using coarse mask (Schoenecker 2008)

Volume censoring | n/a

Statistical modeling & inference

Model type and settings | Voxel-wise mass univariate approach, k-fold cross validation, permutation tests

Effect(s) tested | Voxel-wise spatial correlations and subsequent correlations with clinical hemiscore improvements

Specify type of analysis: ☒ Whole brain ☐ ROI-based ☐ Both

Statistic type for inference | Voxel-wise, k-fold

(See Eklund et al. 2016)

Correction | n/a

Models & analysis

n/a	Involved in the study
☐	☒ Functional and/or effective connectivity
☒	☐ Graph analysis
☒	☐ Multivariate modeling or predictive analysis

Functional and/or effective connectivity | Pearson correlation
